# Unveiling the Unexpected: Co‐Occurrence of Brain Tumor and Spine Pathology Revealed After Spinal Surgery

**DOI:** 10.1155/crnm/6617454

**Published:** 2026-01-12

**Authors:** Shih-Hsiang King, Chih-Ju Chang, Jing-Shan Huang, Foot-Juh Lian

**Affiliations:** ^1^ Division of Neurosurgery, Department of Surgery, Cathay General Hospital, Taipei City, 106, Taiwan, cgh.org.tw; ^2^ Department of Neurosurgery, Hsinchu Cathay General Hospital, Hsinchu, 300, Taiwan, cgh.org.tw; ^3^ Center for Liberal Education, Minghsin University of Science and Technology, Hsinchu County, Xinfeng, 304001, Taiwan, must.edu.tw; ^4^ Division of Neurosurgery, Department of Surgery, Sijhih Cathay General Hospital, New Taipei City, 221, Taiwan, cgh.org.tw; ^5^ Department of Medicine, School of Medicine, Fu Jen Catholic University, New Taipei City, 24205, Taiwan, fju.edu.tw

**Keywords:** brain tumor, cervical radiculomyelopathy, co-occurrence

## Abstract

Cervical spondylotic myelopathy (CSM) is a common cause of spinal cord dysfunction. Because its symptoms may resemble those of intracranial tumors, patients can be misdiagnosed and undergo inappropriate spinal procedures. We describe three patients initially treated with cervical decompression under the impression of CSM. In each case, neurological deficits failed to improve, or even progressed, despite adequate surgery. Further investigation with brain MRI disclosed large meningiomas located in the frontoparietal or parasagittal regions. All tumors were completely resected, pathology confirmed WHO Grade I meningioma, and the patients showed meaningful neurological recovery. These observations remind us that neurological findings must be interpreted in parallel with cervical imaging. A brain MRI should be obtained whenever clinical features are disproportionate to spinal pathology, extend beyond the usual pattern of myelopathy, or remain unresolved after decompression.

## 1. Introduction

The presentation and diagnosis of cervical spondylotic myelopathy (CSM) can be challenging, as signs and symptoms can vary significantly among patients [1]. Common manifestations include gait spasticity and upper extremity numbness, along with impaired fine motor control of the hands [2]. Differential diagnoses that must be considered include brachial plexus neuropathies, thoracic outlet syndrome, shoulder pathology, spinal cord tumors, and brain tumors [1]. Brain tumors, in particular, may present with a wide spectrum of manifestations, ranging from asymptomatic lesions to seizures, persistent headache, cranial nerve deficits, localized neurological deficits, and behavioral abnormalities, and sometimes can mimic those of CSM [3]. The coexistence of CSM and brain tumors, particularly when presenting similar symptoms, makes it difficult to diagnose correctly and choose the right treatment. The approach for certain pathologies should be conducted through the brain cortex, corticospinal tract, anterior horn cells, spinal nerve roots, peripheral nerves, neuromuscular junctions, and ultimately the muscles. In our report, we present three patients initially treated with cervical spine surgery under the impression of CSM, later diagnosed with large meningiomas. These cases underscore the importance of distinguishing between these two pathologies, which initially presented with similar symptoms.

## 2. Case Presentation

### 2.1. Case 1

**Figure 1:**
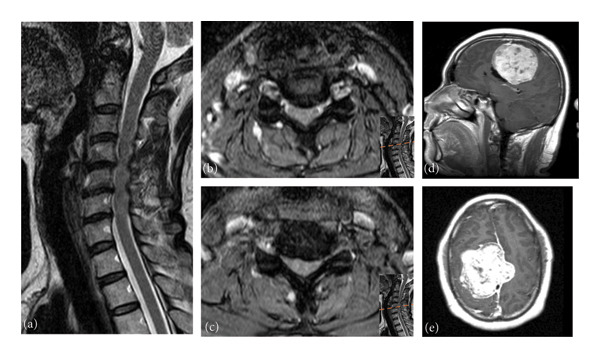
Case 1: Images of cervical magnetic resonance imaging. (a) Sagittal view showing degeneration and ventral spinal cord compression at C4/5, C5/6, and C6/7. (b) Axial views of C4/5. (c) Axial view of C5/6. (d) Sagittal view of brain MRI. (e) Axial view of brain MRI revealed a huge lobulated and well‐defined brain tumor situated in the right frontoparietal parafalcine area, contributing to midline shifting and focal edema.

This 39‐year‐old man presented to our hospital with persistent bilateral forearm pain, tightness, and clumsy walking, diagnosed with CSM with a C4‐5‐6 herniated intervertebral disc (Figure 1). He underwent C3‐6 laminoplasty for decompression of cervical stenosis.

Following 3 years of postoperative follow‐up, the patient reported substantial improvement in symptoms. However, persistent weakness and radiating pain in the left upper limb, as the primary concerns, did not fully recover as compared to his baseline. Intermittent headaches were also noted. Neurological examination revealed paresthesia, arm pain, and muscle weakness (Grade 4), with normal deep tendon reflexes (DTRs). Bilateral hand motor and sensory nerve conduction studies were within normal limits. Brain magnetic resonance imaging (MRI) with gadolinium unveiled a lobulated and well‐defined brain tumor situated in the right frontoparietal parafalcine area, contributing to midline shifting and focal edema (Figure 1). Craniotomy was performed with gross total removal. Pathological analysis confirmed a mixed‐type meningioma of meningothelial and fibrous components, World Health Organization (WHO) Grade I.

Postoperatively, after gross total tumor removal and participation in rehabilitation programs, the patient demonstrated remarkable improvements. Muscle power in both upper limbs increased from Grade 4 to Grade 5. Significant enhancements were observed in functional aspects, including standing balance and gait, allowing the patient to walk without the help of any device.

### 2.2. Case 2

**Figure 2:**
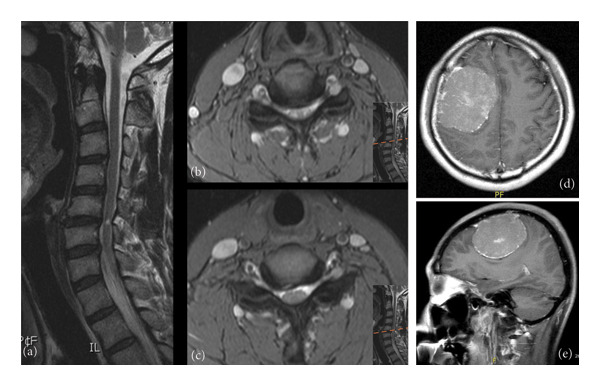
Case 2: Images of cervical magnetic resonance imaging. (a) Sagittal view showing disc bulging with canal stenosis at the C4–7 level. (b) Axial views of C4/5. (c) Axial view of C5/6. (d) Sagittal view of brain MRI. (e) Axial view of brain MRI revealed a well‐defined extra‐axial brain tumor measuring 7.5 cm in the right high frontal convexity area with a significant mass effect.

A 44‐year‐old man presented with neck pain, paresthesia, and mild motor weakness (Grade 4) in the left limbs, along with normal DTRs in the upper extremities (2+) and hyperreflexia in the lower limbs (4+). An MRI of the cervical spine revealed disc bulging with canal stenosis at the C4‐7 level, leading to the diagnosis of CSM (Figure 2). He underwent C4‐6 open‐door laminoplasty with partial laminectomy at C3 and C7. Despite the surgical intervention, the patient continued to experience persistent left limb weakness and an unsteady gait, prompting readmission 3 months later. Subsequent evaluation of the brain was initiated due to ongoing hemiparesis, revealing a well‐defined extra‐axial brain tumor measuring 7.5 cm in the right high frontal convexity area with a significant mass effect (Figure 2). The patient underwent a Simpson Grade 2 tumor removal, and the pathology report confirmed a mixed‐type meningioma comprising meningothelial and fibrous components, WHO Grade I. After surgery, left‐sided paresis improved (Grade 4– Grade 5) and gait imbalance resolved. He was discharged with a satisfying outcome and continued follow‐up in the outpatient department.

### 2.3. Case 3

**Figure 3:**
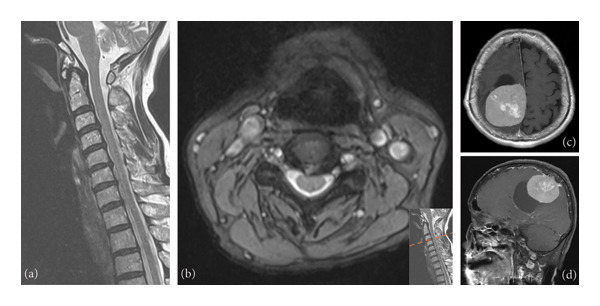
Case 3: Images of cervical magnetic resonance imaging. (a) Sagittal view showing disc bulging at C3‐4, C4‐5, and C5‐6 with mild cord compression. (b) Axial views of C3‐4. (c) Sagittal view of brain MRI. (d) Axial view of brain MRI revealed a sizable 7.0‐cm lesion characterized by cystic formation in the right parietal region. This lesion was causing compression of the cortex surrounding the right central sulcus, accompanied by edema extending into the subcortical white matter.

A 64‐year‐old woman presented with a 1‐year history of left leg numbness and weakness. Cervical imaging showed C3‐4 disc herniation indenting the anterior spinal cord without foraminal stenosis (Figure 3). The patient received evaluations at a local hospital and underwent C3‐4 anterior cervical discectomy and fusion. Unfortunately, her condition deteriorated. She reported persistent left upper and lower limb paresthesia, accompanied by muscle weakness after the surgical intervention. Neurological examination demonstrated hyperreflexia of the left knee jerk and left lower leg weakness (Grade 3). Given the clinical presentation of a possible brain lesion, a brain MRI was conducted, which revealed a sizable 7.0‐cm lesion characterized by cystic formation in the right parietal region. This lesion was causing compression of the cortex surrounding the right central sulcus, accompanied by edema extending into the subcortical white matter (Figure 3). Under the impression of a brain tumor, the patient underwent surgical intervention of a Simpson Grade 2 tumor removal in our department. After surgery, the patient had mild residual paresthesia but significant improvement in hemiparesis. Notably, no new neurological deficits developed postoperatively. Subsequent imaging showed no new pathological findings. The patient’s stable clinical condition allowed discharge with outpatient follow‐up.

## 3. Discussion

The coexistence of CSM and intracranial tumors is extremely rare. Only isolated case reports and small series have been published, and no reliable incidence or prevalence data are available. The existing literature mentions only a few cases of concurrent brain tumor and degenerative cervical spine pathology, with the majority presenting as cervical radiculopathy [1, 3–5] (Table 1). These cases consistently illustrate the diagnostic challenge posed by overlapping symptoms and the risk of attributing new neurological deficits solely to cervical disease. To avoid redundancy, the detailed case descriptions from prior publications are summarized in Table 1, with key clinical lessons highlighted.

**Table 1:** The summary of published cases with concurrent brain tumor and degenerative cervical spine pathology.

Ref.	Author/year	Age	Gender	Cervical pathology	Brain pathology	Significance
[5]	Clar and Cianca, 1998	55	Male	C5‐6, C6‐7 intervertebral disc degeneration	Right precentral gyrus glioblastoma	Early diagnosis and the potential benefits of prolonged life and improved QoL through symptom reduction with early intervention

[4]	Khalatbari et al., 2008	56	Female	C5‐6 intervertebral disc degeneration	Left parietal convexity meningioma	Underscores the surgical decision‐making challenges posed by coexisting lesions that may cause similar symptoms

[3]	Huang et al., 2014	54	Male	C3‐4, C4‐5, and C5‐6 HIVD	Right frontoparietal meningioma	Difference between typical features and the progression of clinical symptoms, a more cautious neurological examination might have identified the brain lesion earlier

[1]	So et al., 2022	48	Male	C6–7 disc degeneration	Left frontoparietal meningioma	Emphasized the need for clinicians to consider the possibility of a new brain lesion even if previous brain MRI appeared normal
		58	Male	C5–6 disc degeneration and foraminal narrowing	Right precentral gyrus metastasis	

—	Current Case 1	39	Male	CSM and C5‐6 HIVD	Right frontoparietal meningioma	Focus on clinical symptoms, signs, and imaging evidence to identify indicators in the diagnostic process for CSM to differentiate it from other confusing diagnoses
—	Current Case 2	44	Male	CSM with C4‐7 canal stenosis	Right frontal convexity meningioma	
—	Current Case 3	64	Female	CSM with C3‐4 HIVD	Right parietal parasagittal meningioma	

Abbreviations: CSM = cervical spondylotic myelopathy, HIVD = herniated intervertebral disc, MRI = magnetic resonance imaging, QoL = Quality of life.

In our three cases, the patients typically present with symptoms of radiculomyelopathy. If the manifestation of a brain tumor aligns with that of degenerative cervical myelopathy, distinguishing between them becomes particularly challenging, as both are upper motor neuron lesions. This similarity in presentation and neurological examination findings, compounded by coincidental spondylotic changes in imaging, can lead to misdiagnoses and inappropriate treatment. We aim to focus on clinical symptoms, signs, and imaging evidence to identify subtle indicators in the diagnostic process for degenerative cervical myelopathy and to differentiate it from other confusing diagnoses, particularly giant brain tumors, which are generally considered too critical to be overlooked.

CSM arises from a combination of static and dynamic mechanical factors and exhibits a spectrum of clinical symptoms [2]. Patients with CSM often initially experience paresthesia in one or more extremities. As the condition progresses, they may develop numbness, difficulty with fine motor skills (such as buttoning shirts or writing), and gait disturbances characterized by a sense of imbalance or leg heaviness, resulting in an unsteady gait [2]. In advanced stages, some individuals may also experience bowel or bladder dysfunction. While there is no pathognomonic sign for CSM, Munro et al. [6] noted that 59.1% of CSM patients first reported neck, shoulder, or upper limb sensory symptoms, 84.2% experienced poor balance, and over 70% presented with distal motor problems, including issues with grip strength or dexterity.

In contrast, brain tumors produce neurological deficits depending on their cortical location [7]. Unlike the distal problems commonly associated with CSM, tumors located in the parasagittal and high convexity regions more frequently impact motor functions of the trunk and proximal upper limbs according to the 2D surface displayed on the lateral aspect of the central frontoparietal cortex reported by Catani [8]. When a tumor affects the somatosensory cortex, it can lead to sensory deficits such as numbness, tingling, or loss of sensation in the corresponding area of the homunculus. The size and growth rate of the tumor can intensify these symptoms; as the tumor enlarges, it increases pressure on adjacent brain tissues, further disrupting normal neurological functions.

Practical red flags suggesting intracranial pathology before spine surgery include the following:

1. Exam‐imaging incongruity (severity or laterality of deficits not explained by cervical findings);
2. Features beyond a typical myelopathic pattern, such as hemiparesis, new or progressive headache, seizures, cognitive/behavioral change, visual symptoms, or papilledema;
3. Failure to improve as expected after technically adequate cervical decompression.

MRI is a crucial tool in diagnosing CSM and often serves as a key determinant in the decision to pursue surgical intervention. Therefore, accurately correlating a patient’s symptoms with MRI findings is of paramount importance. Research in the field indicates that approximately 20% of healthy individuals (or those with unrelated symptoms) exhibit spinal cord compression on MRI, which increases to 86% among populations exhibiting myelopathic features [9]. In other words, one in five asymptomatic individuals may show cord compression on MRI, while in a group of 10 people with myelopathic symptoms, not all may have symptoms caused by cord compression. Cao et al. [10] highlight that cervical segmental instability, high intramedullary signal on T2‐weighted MRI, and the Torg ratio are three distinguishing factors between patients with asymptomatic and symptomatic CSM in cases of mild to moderate cervical spinal cord compression. These findings align well with the understood etiology of cervical myelopathy, which includes a combination of static, dynamic mechanical, and ischemic factors. This insight is invaluable in evaluating MRIs of patients with myelopathic symptoms, providing critical considerations for diagnosis.

Taken together, our cases illustrate that giant brain tumors may coexist with degenerative cervical myelopathy and remain unrecognized until after spine surgery. Thorough neurological examination and strict correlation between clinical symptoms and cervical imaging are essential before operating. Red flags that should prompt brain MRI include hemiparesis, headache, seizures, progressive imbalance, or any exam‐imaging incongruity. Based on these lessons, in our current practice, we now obtain a brain MRI before surgery in patients with atypical features or unexplained deficits. Such vigilance can prevent unnecessary spinal procedures and ensure timely treatment of intracranial disease.

**Nomenclature**
CSM: Cervical spondylotic myelopathy
MRI: Magnetic resonance imaging
DTR: Deep tendon reflex
WHO: World Health Organization

## Consent

Written informed consent was obtained from the patients for publication of this case report.

## Conflicts of Interest

The authors declare no conflicts of interest.

## Funding

No funding was received for this manuscript.

## Data Availability

The data supporting the findings of this case report are available from the corresponding author upon reasonable request. Because of patient privacy and ethical restrictions, raw clinical data are not publicly available.
